# Germline mutations in a DNA repair pathway are associated with familial colorectal cancer

**DOI:** 10.1172/jci.insight.148931

**Published:** 2021-09-22

**Authors:** Pingping Xu, Danfeng Sun, Yaqi Gao, Yi Jiang, Ming Zhong, Gang Zhao, Jinxian Chen, Zheng Wang, Qiang Liu, Jie Hong, Haoyan Chen, Ying-Xuan Chen, Jing-Yuan Fang

**Affiliations:** 1 State Key Laboratory for Oncogenes and Related Genes, Key Laboratory of Gastroenterology & Hepatology, Ministry of Health, Division of Gastroenterology and Hepatology, Shanghai Institute of Digestive Disease,; 2 Department of Surgery, and; 3 Department of Pathology, Renji Hospital, School of Medicine, Shanghai Jiao Tong University, Shanghai, China.

**Keywords:** Gastroenterology, Genetics, Cancer, Cell cycle, DNA repair

## Abstract

Aiming to identify rare high-penetrance mutations in new genes for the underlying predisposition in familial colorectal cancer (CRC), we performed whole-exome sequencing in 24 familial CRCs. Mutations in genes that regulate DNA repair (*RMI1*, *PALB2*, *FANCI)* were identified that were related to the Fanconi anemia DNA repair pathway. In one pedigree, we found a nonsense mutation in *CHEK2*. *CHEK2* played an essential role in cell cycle and DNA damage repair. Somatic mutation analysis in *CHEK2* variant carriers showed mutations in *TP53*, *APC*, and *FBXW7*. Loss of heterozygosity was found in carcinoma of *CHEK2* variant carrier, and IHC showed loss of Chk2 expression in cancer tissue. We identified a second variant in *CHEK2* in 126 sporadic CRCs. A KO cellular model for *CHEK2* (*CHEK2*^KO^) was generated by CRISPR/Cas9. Functional experiments demonstrated that *CHEK2*^KO^ cells showed defective cell cycle arrest and apoptosis, as well as reduced p53 phosphorylation, upon DNA damage. We associated germline mutations in genes that regulate the DNA repair pathway with the development of CRC. We identified *CHEK2* as a regulator of DNA damage response and perhaps as a gene involved in CRC germline predisposition. These findings link CRC predisposition to the DNA repair pathway, supporting the connection between genome integrity and cancer risk.

## Graphical abstract

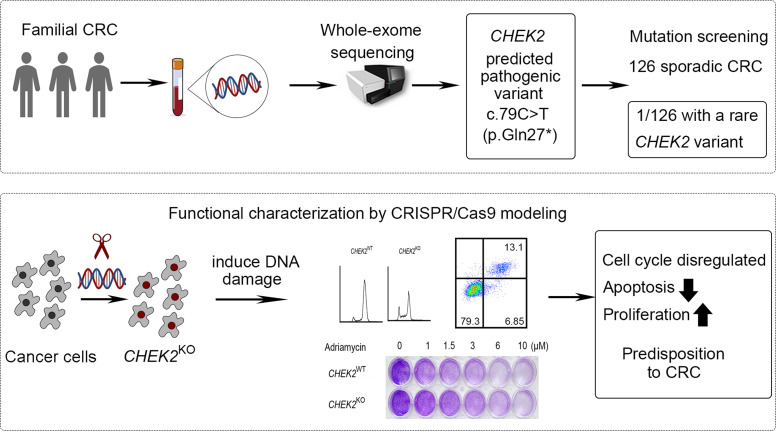

## Introduction

Colorectal cancer (CRC) is a common disease with a high mortality rate in the world. Germline predisposition and environmental factors affect CRC susceptibility. Importantly, the inherited germline contribution is known to influence about 12%–35% of all cases (1, 2). However, only 5%–7% of CRC cases are caused by germline mutations in genes that are responsible for Mendelian cancer syndromes. Lynch syndrome and familial adenomatous polyposis (FAP) are the most frequent forms of Mendelian CRC syndromes. Classic hereditary CRC syndromes are mainly due to germline mutations in *APC*, *MUTYH*, and the mismatch repair genes (*MSH2*, *MSH6*, *PMS2*, *MLH1*; refs. 3, 4).

In addition to hereditary forms, around 30% of CRC cases also present familial aggregation — but with an unknown inherited cause. The hypothesis of rare high-penetrance mutations in genes yet to be discovered is a very likely explanation for the underlying predisposition in a portion of these familial CRC cases. Therefore, past efforts have been made in these familial CRC cases, and next-generation sequencing technologies added a new unbiased approach to facilitate the identification of new genes responsible for predisposition to CRC. New candidate genes related to CRC have been found, such as *POLD1*, *POLE* (encoding DNA polymerases), *NTHL1* (encoding a base-excision repair protein), *MSH3*, *GREM1*, *RNF43*, *RSP20*, *MLH3*, *FAF1*, and *MCM8* (5–14). However, a large part of the heritability of colorectal adenomatous polyposis and CRC remains unexplained and is widely postulated to be enshrined in unidentified, rare variants.

With the aim of identifying new hereditary CRC genes, we performed whole-exome sequencing (WES) in patients with familial CRC. Our final goal is to facilitate genetic counsel and to be able to correctly address prevention strategies in these families.

## Results

### Clinical characteristics and germline sequencing results.

We examined 24 individuals from 21 families with CRC or advanced colorectal adenoma (CRA) by WES (Supplemental Tables 1 and 2; supplemental material available online with this article; https://doi.org/10.1172/jci.insight.148931DS1). All individuals had strong disease aggregation compatible with an autosomal-dominant pattern of inheritance. To exclude known Mendelian cancer syndromes, all individuals were screened for mutations in *APC*, *MUTYH*, and mismatch repair genes (*MSH2*, *MSH6*, *PMS2*, *MLH1*) before WES analysis. All individuals were tested without mutations in known hereditary CRC, including FAP or Lynch syndrome.

WES was performed in all individuals with a mean coverage of 86× (Supplemental Table 3). Germline WES data analysis was selected for only vary rare variants (<0.1%) producing a loss of function (LOF) or variants located on genes with a function compatible with cancer development (7, 8, 10).

**Figure 1:**
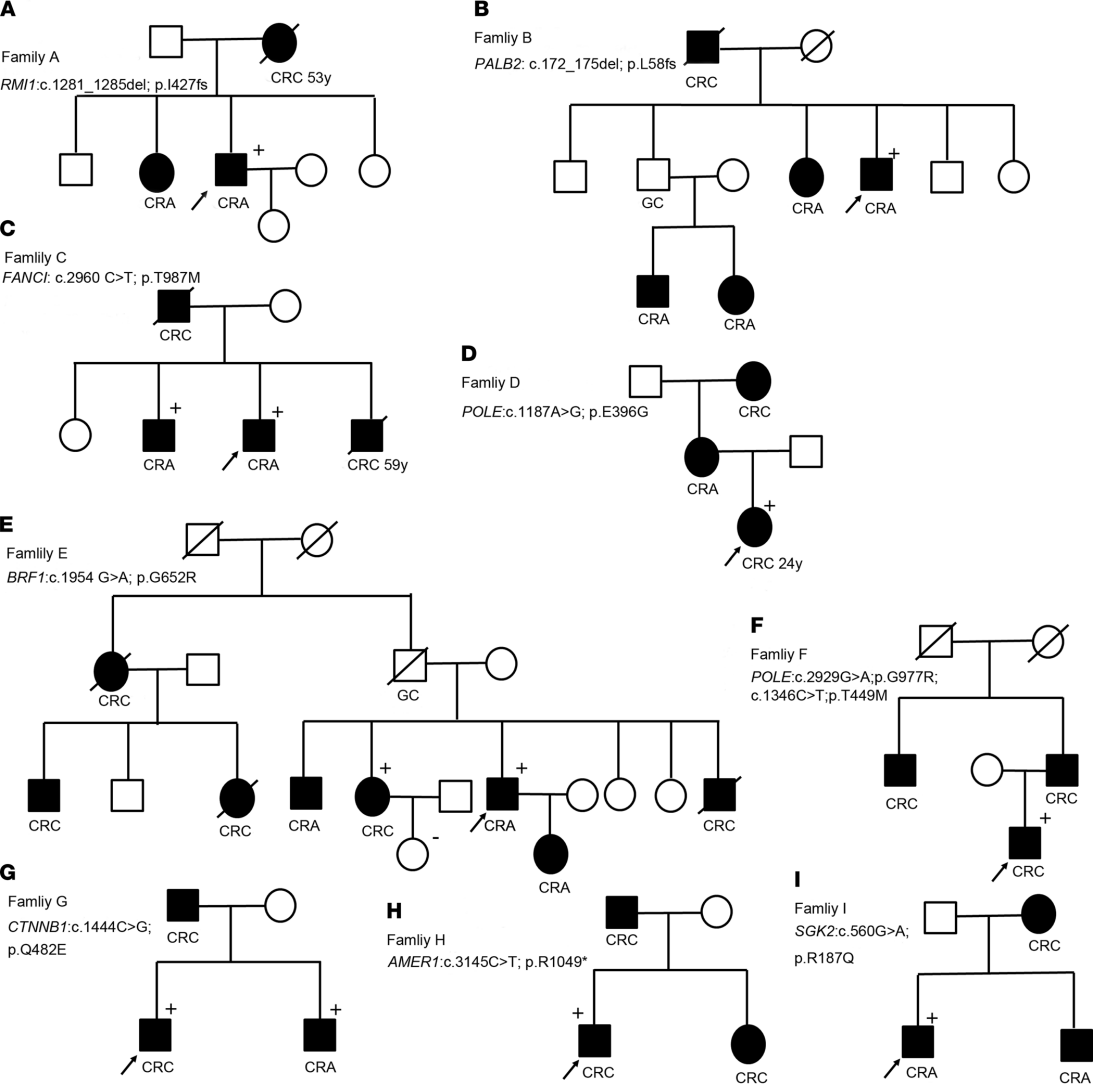
Pedigrees of study participants with strong family histories of CRC and the identifiable mutations. (**A**–**I**) Squares indicate male family members, and circles represent female members. A slash through a symbol indicates that the family member has died. Filled symbols indicate those affected by colorectal cancer or advanced colorectal adenomas. (+), mutation carrier; (–), nonmutation carrier. The proband is indicated by an arrow. The clinical details, including the features of the additional relatives and the number of tumors in those families, are shown in Supplemental Table 13.

After filtering the variants, we detected LOF mutations in 6 different genes (*RMI1*, *PALB2*, *FANCI*, *AMER1*, *CTNNB1*, *SGK2*; Figure 1 and Table 1). Among this, we found 3 mutations related to the Fanconi anemia (FA) DNA repair pathway (*RMI1*, *PALB2*, *FANCI*; Table 1). We also found 3 mutations in *POLE* in 2 individuals. All variants were verified by Sanger sequencing (Supplemental Figure 1). Additional segregation for these variants was performed when available. Unfortunately, most of family members were not accessible (Figure 1).

**Figure 2:**
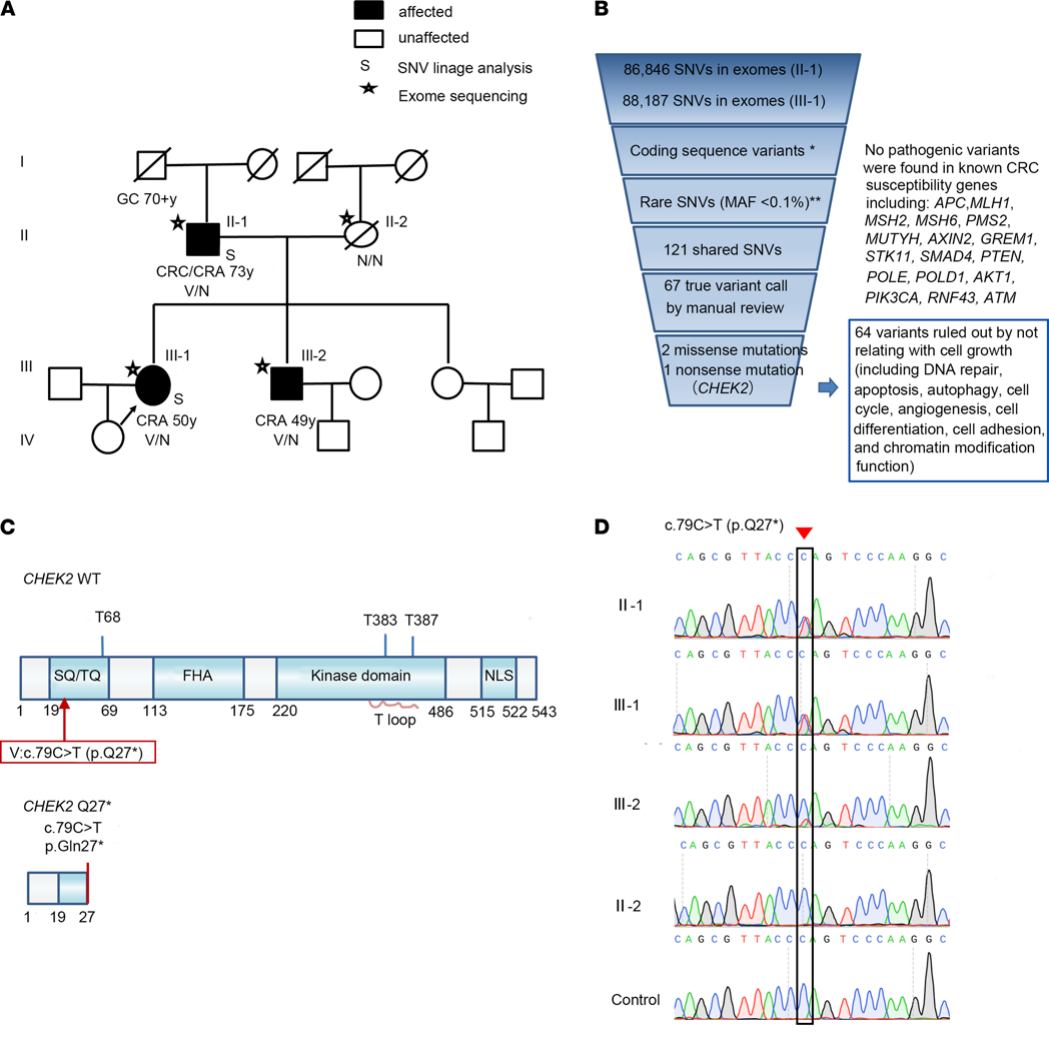
Gene discovery and characterization. (**A**) Pedigree K. Squares indicate male family members, and circles represent female members. A slash through a symbol indicates that the family member has died. Filled symbols indicate a clinically affected family member. The proband is indicated by an arrow. WES analysis was performed on 2 individuals, which are marked by the letter S. The CHEK2_p.Q27* mutant germline allele that was detected by Sanger sequencing is shown below each individual. “V/N” indicates a heterozygous variant carrier, and “N/N” indicates a noncarrier. Age of tumor diagnosis is shown beneath each symbol. (**B**) The filter-based computational algorithm that is used to narrow candidate variants for pedigree K. Single-nucleotide variant, SNV; minor allele frequency, MAF. (**C**) The functional domains of Chk2 and the predicted truncated Chk2 protein that would result from the variant. SQ/TQ indicates the SQ/TQ motif, which is the consensus site for phosphoinositide-kinase–related kinases (PIKKs). FHA indicates the forkhead-associated domain. NLS indicates the nuclear localization signal. (**D**) Sanger sequencing–based validation of the germline CHEK2_p.Q27* mutation in individuals of this pedigree. The red arrowhead and black box indicate the location of the heterozygous substitution of C to T in the mutated locus of CHEK2.

In addition, we found 1 potentially pathogenic *CHEK2* variant in the pedigree K (Figure 2A) that contained 2 individuals (II-1 and III-1) that had been sequenced. In this family, the proband (individual III-1) was first diagnosed with CRA at 50 years old and had recurrently multiple CRAs 2 and 7 years later, with the biggest adenoma being 1.5 × 1.5 cm and tubular. The proband’s father (individual II-1) was diagnosed with sigmoid colon adenocarcinoma at 73 years old due to ileus and underwent a colectomy. He was followed by colonoscopy every 6 or 12 months. At least 33 polyps were detected in his colon, with the biggest polyp being 2.5 × 2.3 cm, and they were predominantly advanced adenomas. The proband’s brother (individual III-2) was first diagnosed with multiple CRAs at 49 years old. The proband’s grandfather was diagnosed with gastric cancer at 70 years old and died several months after surgery. All the known members of this pedigree were not diagnosed with breast cancer.

We searched the sequences for genes that harbored damaging variants. After performing variant filtration and quality control, we filtered annotated variants and identified 3 heterozygous variants in 3 genes (Supplemental Table 4; Supplemental Table 5, A and B; Figure 2B; and Supplemental Table 6 for analysis flow chart). Two of these variants were missense mutations in *CHD8* and *MOB1B*, but the mutations were not found in the key domain of the protein. The third variant contained the highest Combined Annotation-Dependent Depletion (CADD) score (CADD score of 35) and was the only LOF variant. This *CHEK2* (GenBank: NM_001005735) variant (hg19chr22: g.29130631 C>T) (ClinVar accession SCV001754535; https://www.ncbi.nlm.nih.gov/clinvar/) is predicted to cause a premature stop code in the SQ/TQ motif at position 27 (p.Gln27*, also named as p.Q27*; Figure 2C). Sanger sequencing further confirmed the variant in individuals II-1 and III-1 (Figure 2D), as well as another affected family member (III-2; Figure 2D). Segregation analysis revealed the presence of the same germline *CHEK2* variant in affected family members (II-1, III-1, III-2) — where 1 individual exhibited CRC and multiple CRAs (individual II-1), and 2 individuals exhibited multiple CRAs (individual III-1 and III-2; Figure 2A). The 1000 Genomes Project, Exome Aggregation Consortium (ExAC), and the Genome Aggregation Database (gnomAD) do not contain this variant, confirming the identified mutation as a rare event (ftp-trace.ncbi.nih.gov/1000genomes; https://gnomad.broadinstitute.org/). Hence, we consider this mutation (*CHEK2* p.Q27*) as a good candidate for CRA/CRC.

### Tumor analysis.

Furthermore, we examined 7 tumors (6 adenomas and 1 carcinoma) from 3 carriers of the *CHEK2*_p. Q27* variant using WES analysis (Supplemental Tables 7 and 8) to explore somatic mutations in tumor tissues. All tumors were screened for *KRAS* and *BRAF* driver mutations, and a total of 83 cancer-related genes were screened, including *APC*, *CTNNB1*, *PIK3CA*, and *FBXW7* (Supplemental Table 9 and refs. 15, 16). All tumors were microsatellite stable (MSS; Table 2 and Supplemental Table 8). As shown in Table 2, somatic mutations in tumor tissues were mainly found in *APC*, *TP53*, and *FBXW7*. For *APC*, all mutations were LOF variants (nonsense and frameshift). There was also a missense mutation in *TP53* (NM_001126112:c.742C>T, p.R248W) in the carcinoma tissues, suggesting that *TP53* may be involved in the colorectal tumorigenesis in this case. The *TP53* mutation was located in the DNA-binding domain, which is an important structural domain. However, mutations were not found in other pathogenic genes, including *CTNNB1*, *KRAS*, and *BRAF*.

**Figure 3:**
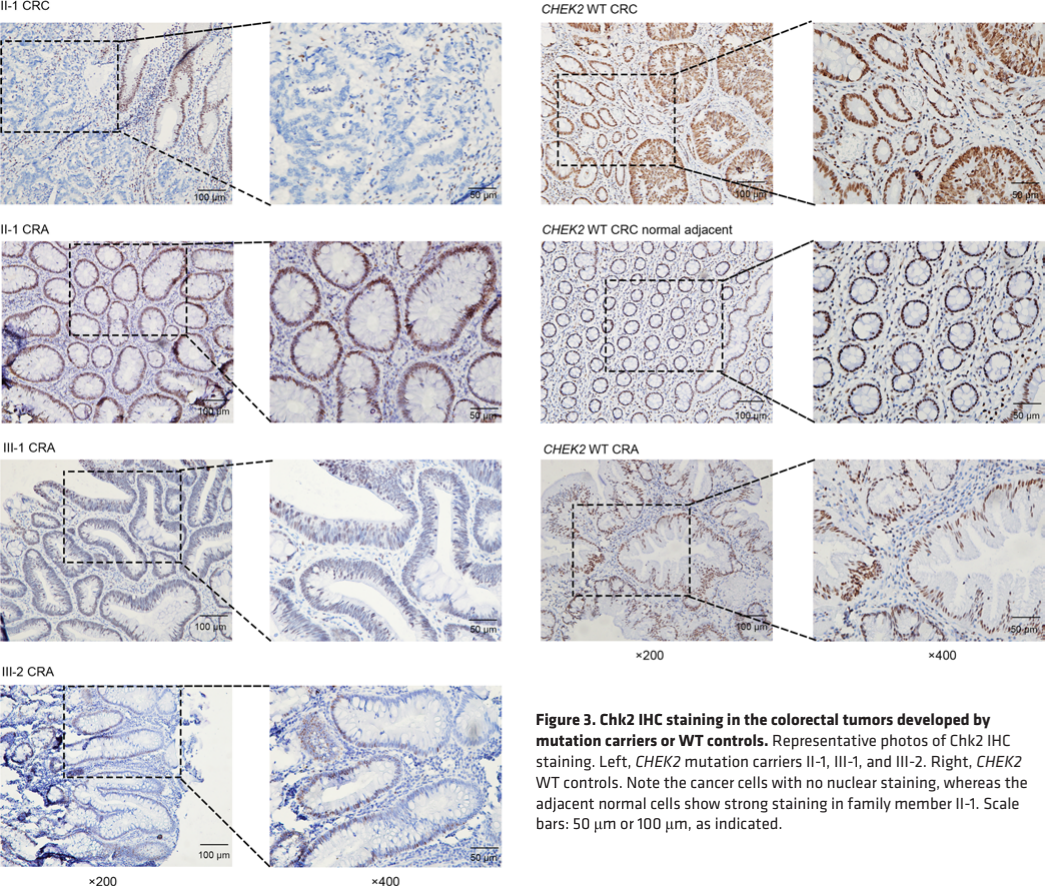
Chk2 IHC staining in the colorectal tumors developed by mutation carriers or WT controls. Representative photos of Chk2 IHC staining. Left, *CHEK2* mutation carriers II-1, III-1, and III-2. Right, *CHEK2* WT controls. Note the cancer cells with no nuclear staining, whereas the adjacent normal cells show strong staining in family member II-1. Scale bars: 50 μm or 100 μm, as indicated.

Loss of heterozygosity (LOH) involving the germline WT allele was found in the cancer tissue of II-1 (Supplemental Table 8). However, IHC staining of Chk2 in the adenoma tissues of 3 *CHEK2* variant carriers including individual II-1 revealed nuclear expression of the protein (Figure 3). It is noteworthy that in II-1’s cancer tissue, Chk2 expressed in adjacent normal cells but not in cancer cells, indicating somatic inactivation of the WT allele in cancer cells. This is consistent with the *CHEK2* LOH in cancer tissue detected by WES (Supplemental Table 8).

### CRISPR/Cas9 CHEK2^KO^ modeling.

**Figure 4:**
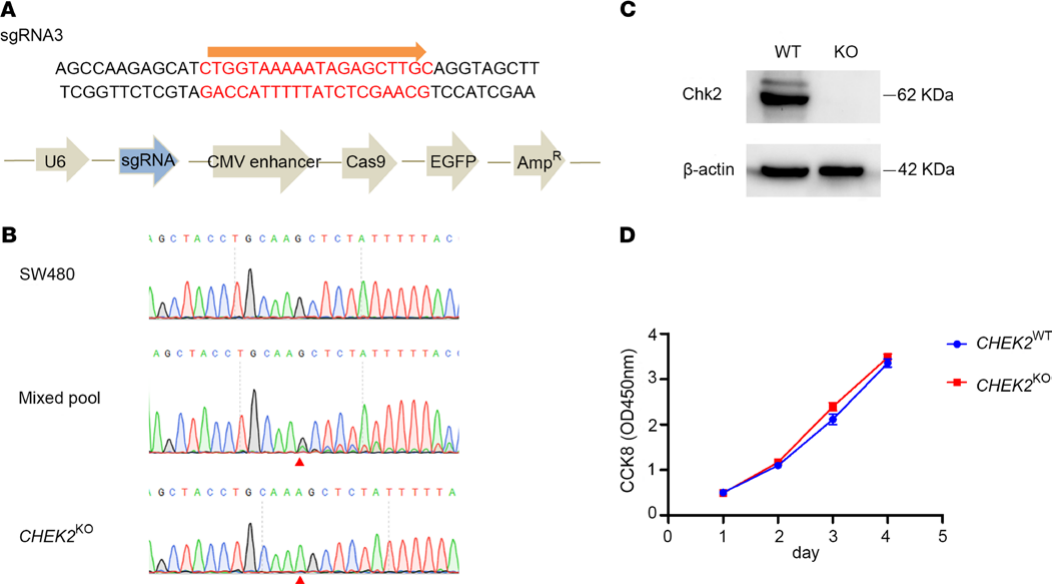
CRISPR/Cas9-mediated *CHEK2* gene inactivation in CRC cell lines. (**A**) CRISPR-directed *CHEK2* inactivation was designed targeting exon 10. (**B**) The aberrant sequence signal after the expected break site of the edited cell pool by Sanger sequencing (red arrowhead). (**C**) Chk2 protein level of the selected *CHEK2*^KO^ clones assessed by Western blot. (**D**) Proliferation of *CHEK2*^WT^ and *CHEK2*^KO^ cells detected by CCK8 after cultured for the indicated time. Data are expressed as mean ± SEM (*n =* 6), nonparametric Mann–Whitney *U* test.

As shown in pedigree K, we found that the *CHEK2* variant (p.Q27*) led to a premature stop at amino acid 27 and resulted in the loss of normal protein structure and function in Chk2. Using CRISPR/Cas9 technology, we generated *CHEK2* knock-out (*CHEK2*^KO^) SW480 cells to reproduce the LOF mutant and further investigate the physiological role of Chk2 (Figure 4A). According to bioinformatic CRISPR prediction tools, a single-guide RNA (sgRNA) targeting the tenth exon was selected. The genotype of *CHEK2*^KO^ clones was determined by Sanger sequencing (Figure 4B). The complete loss of Chk2 production in *CHEK2*^KO^ cells was confirmed by Western blot analysis (Figure 4C). We found that there was no influence on cell proliferation after *CHEK2* KO (Figure 4D). This indicated that this CRISPR cell model can be used to explore the functional characterization of *CHEK2*.

### Functional characterization of germline variants.

**Figure 5:**
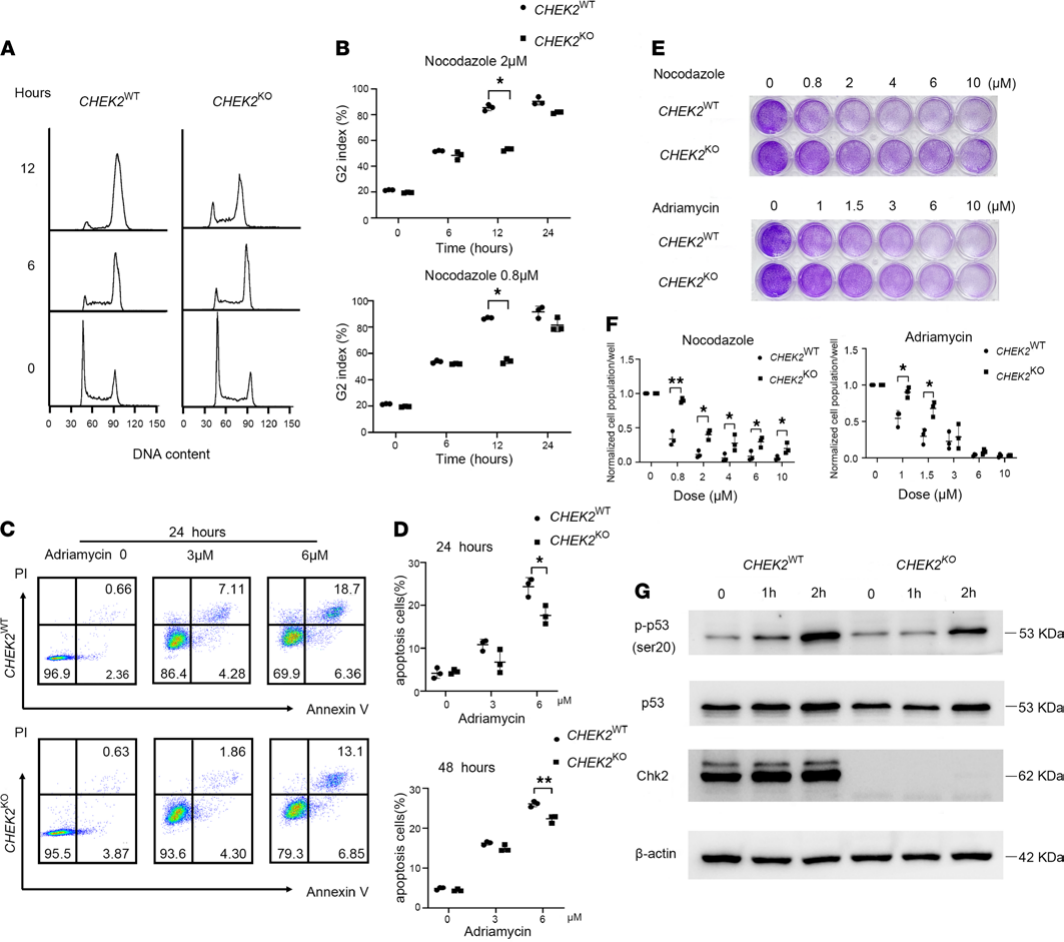
Failure of maintenance of nocodazole-induced G2 arrest and impaired DNA damage–induced apoptosis in *CHEK2*^KO^ cells. (**A**) Kinetics of cell cycle progression of *CHEK2*^WT^ and *CHEK2*^KO^ cells after nocodazole treatment (2 μM) for 0, 6, and 12 hours. (**B**) The percentage of cells that arrest at G2 after nocodazole treatment at different time points and concentrations. Data are expressed as mean ± SEM (*n =* 3). *P < 0.05, nonparametric Mann–Whitney *U* test. (**C**) Cellular apoptosis analysis by flow cytometry in *CHEK2*^WT^ and *CHEK2*^KO^ cells treated with different concentrations of Adriamycin after 24 hours. (**D**) The percentage of apoptotic cells calculated by flow cytometry from **C**. Data are expressed as mean ± SEM (*n =* 3). *P < 0.05, **P < 0.01, nonparametric Mann–Whitney test. (**E**) Cell viability detected by a crystal violet assay after cells were treated with different concentrations of nocodazole or Adriamycin. (**F**) Normalized cell population/well calculated by ImageJ software (NIH) from **E**. Data are expressed as mean ± SEM (*n =* 3). *P < 0.05, nonparametric Mann–Whitney *U* test. (**G**) Protein levels of pan-p53, phosphorylated p53 (serine 20), and Chk2 in CHEK2^WT^ and CHEK2^KO^ cells after Adriamycin treatment for the indicated times.

*CHEK2*^KO^ cells show a defect in G2 cell cycle arrest after DNA damage*.* Given that Chk2 is activated in response to DNA damage and may regulate cell cycle arrest, we treated *CHEK2*^WT^ and *CHEK2*^KO^ SW480 cells with nocodazole, a microtubule-disrupting agent that traps cells in mitosis. When cells were treated with nocodazole at different concentrations, the expression of phospho-γH2AX was elevated when compared with the untreated cells, and DNA damage was induced (Supplemental Figure 2A). When cells were treated with nocodazole for 6 hours, the cell cycle was arrested and about 50% of *CHEK2*^WT^ and *CHEK2*^KO^ cells were arrested in G2 phase (Figure 5, A and B). After 12 hours, more *CHEK2*^KO^ cells entered G1 and S phases relative to *CHEK2*^WT^ cells. These results indicated that there was a defect in G2 cell cycle arrest in *CHEK2*^KO^ cells after DNA damage.

### CHEK2^KO^ cells were resistant to DNA damage–induced apoptosis and influence p53 phosphorylation during DNA damage.

If damaged DNA cannot be repaired, cells can initiate apoptosis, which may also be regulated through the Chk2 kinase. To investigate the role of Chk2 in apoptosis, Adriamycin (which can induce double-stranded DNA breaks) was used to treat *CHEK2*^WT^ and *CHEK2*^KO^ cells. When *CHEK2*^WT^ cells were treated with Adriamycin (6 μM), around 69% and 57% of cells were viable after 24 and 48 hours, respectively (Figure 5, C and D, and Supplemental Figure 2B). With the same concentration of Adriamycin, around 80% and 71% of *CHEK2*^KO^ cells were viable after 24 and 48 hours, respectively (Figure 5, C and D, and Supplemental Figure 2B). There was a significantly lower percentage of apoptotic cells in Adriamycin-treated *CHEK2*^KO^ cell samples compared with Adriamycin-treated *CHEK2*^WT^ cell samples (Figure 5, C and D). The crystal violet assay also confirmed that Adriamycin-treated *CHEK2*^KO^ cells had higher cell viability than Adriamycin-treated *CHEK2*^WT^ cells. As the concentration of Adriamycin increased, the difference in cell survival between *CHEK2*^WT^ and *CHEK2*^KO^ cells became apparent (Figure 5, E and F). *CHEK2*^KO^ cells were also resistant to apoptosis induced by other agents that induce DNA damage, such as nocodazole (Figure 5E and Supplemental Figure 2, C and D). This result indicates that *CHEK2*^KO^ cells were resistant to DNA damage-induced apoptosis.

It has been reported that Chk2 regulates cell cycle arrest and apoptosis during the DNA damage response through p53. Chk2 can directly phosphorylate p53 on serine20 to disrupt its association with Mdm2, thus promoting its stability (17–19). To explore the function of Chk2 in p53 regulation, the protein level of p53 after DNA damage was assessed. As shown in Figure 5G, *CHEK2*^WT^ cells expressed high protein levels of phosphorylated p53 on serine 20 when treated with Adriamycin. In contrast, *CHEK2*^KO^ cells had reduced levels of pan-p53 and phosphorylated p53 compared with *CHEK2*^WT^ cells (Figure 5G). This result suggests that Chk2 may influence p53 phosphorylation during DNA damage.

### Screening of the candidate gene variants in an independent cohort.

In order to investigate the frequency of *CHEK2* in sporadic CRC/CRA and normal populations, we genotyped another cohort to screen for the mutation encoding the *CHEK2*_p.Q27* variant. The cohort included 352 individuals of Chinese ancestry with colorectal tumors (including 230 individuals with CRC and 122 individuals with CRA; Supplemental Table 10). For comparison, we genotyped 100 control individuals, who were of Chinese ancestry but had not been diagnosed with polyposis or CRC (Supplemental Table 10). We found no additional cases or no controls that were heterozygotes for the mutation encoding the *CHEK2*_p.Q27* variant, further confirming *CHEK2*_p.Q27* was a rare event.

We screened the entire *CHEK2* coding sequence in the germline DNA from 126 sporadic CRC patients by Sanger sequencing. An additional rare missense variant (NM_001005735:c.766 C>G, p. P256A) was detected in these sporadic cases (Supplemental Figure 1). This identified missense variant was not found in a large population dataset (gnomAD; https://gnomad.broadinstitute.org/)) and was predicted to be potentially pathogenic in silico tools (e.g., SIFT prediction: deleterious) (http://provean.jcvi.org/index.php). However, segregation for the detected genetic variant was not available in the family.

## Discussion

In this study, we identified potential CRC predisposition variants in genes (*RMI1*, *PALB2*, *FANCI*, and *CHEK2*) that regulate the DNA damage response, including the FA DNA repair pathway and the cell cycle.

It is widely held that genomic instability is a prerequisite for cancer formation. Colorectal epithelial cells are recurrently exposed to endogenous and exogenous mutagens and having high turnover rates (20). Therefore, DNA damage repair (DDR) mechanisms are fundamental to maintain the genomic integrity of the cell. The malfunctioning of DDR is strongly associated with carcinogenesis agents. Checkpoint mechanisms serve a major regulatory function in governing the DDR and ensure the coordination of DNA repair proteins, which detect and repair DNA damage to protect cells from genome instability.

FA is an inherited genomic instability disorder that contains bone marrow failure, growth abnormalities, and cancer predisposition. FA patients have chromosome fragility and hypersensitivity to drugs that induce DNA interstrand crosslinks (ICLs; ref. 21). The FA repair pathway is thought to coordinate a complex mechanism that contains elements of 3 classic DNA repair pathways in response to genotoxic insults. These 3 pathways include homologous recombination, nucleotide excision repair, and mutagenic translation synthesis (22). When the FA pathway is impaired, cells are hypersensitive to DNA damage and are unable to successfully repair damaged DNA and cause genome instability. Mutations in the FA proteins lead to a high tumor incidence. Previous studies have indicated that malfunctioned FA genes and proteins have been found to be associated with a variety of cancers (23–27).

Previous studies have found *FANCD2/FANCI*-associated nuclease 1 gene (*FAN1*) mutations in the inherited susceptibility to CRC (28). In our study, by exome sequencing, we identified 3 potential CRC predisposition variants (*RMI1*, *PALB2*, *FANCI*) that were involved in the FA repair pathway.

*RecQ-mediated genome instability protein 1* (*RMI1*) is an essential component of the RMI complex that plays an important role in the processing of homologous recombination. The *RMI1* mutation (NM_024945: c.1281_1285del, p.I427fs) (ClinVar accession SCV001754808) is predicted to cause a premature stop and loss of protein function. It is likely to contribute to genomic instability. It has been reported that the *RMI1* gene polymorphisms were associated with the risk of cancer (29, 30), but further studies are needed to investigate.

Partner and localizer of *BRCA2* (*PALB2*, also termed FA complementation group N [FANCN]) plays a critical role in homologous recombination repair (HRR) through its ability to recruit *BRCA2* and *RAD51* to DNA breaks. Mutations in *PALB2* have been reported in breast cancer, Fanconi anemia subtype FA-D1, and pancreatic cancer (25, 26, 31–33). Previous studies also identify the association between *PALB2* mutations and early-onset CRC (34). In our study, the *PALB2* mutation (NM_024675: c.172_175del, p.L58fs) (ClinVar accession SCV001754809) is predicted to abolish protein function, thus causing the LOF in the HRR. It is likely to cause the impaired FA repair pathway and contribute to tumorigenesis.

FA Complementation Group I (*FANCI*) plays an essential role in the repair of DNA double-strand breaks by homologous recombination. It takes part in the repair of DNA ICLs with *FANCD2*. It has been reported that *FANCI* mutations possibly involved in breast cancer and ovarian cancer susceptibility (27, 35). The *FANCI* mutation (NM_001113378: c.2960C>T, p.T987M) (ClinVar accession SCV001754757) is located inside the *FANCI* solenoid 3 domain. In silico pathogenicity tools predicted it as a possible pathogenic mutation. Because the unique nuclear protein complex that ubiquitinates *FANCD2* and *FANCI* leads to formation of DNA repair structures, it is postulated that they may affect cancer risk in a specific manner.

FA repair pathway plays an important role in maintaining the genome stability; cell cycle regulations also are critical for DDR. In our study, we also identified a rare genetic variant with plausible pathogenicity in the *CHEK2* gene in 3 individuals from 1 family with CRC. A functional characterization of mutation was performed in CRISPR/Cas9 cellular model to further confirm the pathogenicity and involvement in germline predisposition to CRC.

The serine/threonine protein kinase Chk2 encoded by *CHEK2* is activated in response to DNA damage and subsequently regulates downstream effector proteins, including p53, BRCA1, and BRCA2, which are critical for DNA repair, cell cycle regulation, and cellular apoptosis (36, 37). Given the critical role of mitosis in cell survival, defects in cell cycle regulation may lead to abnormal cell division during DNA damage. Thus, germline mutation in *CHEK2* may cause genomic instability and lead to cancer predisposition. Germline *CHEK2* variants were first reported in families with Li-Fraumeni syndrome that lack *TP53* mutations (38). Later, numerous studies reported that *CHEK2* is a multiorgan cancer susceptibility gene, such as breast (39), ovarian (40), prostate (41), and renal cancer (42). Recently, a multicenter case-control analysis using WES provided evidence for germline *CHEK2* LOF variants as new moderate-penetrance variants in testicular germ cell tumors (43). Although it has been reported that *CHEK2* I157T associates with an increased risk of CRC (44), no causal germline mutation of *CHEK2* in CRC has been identified. In our study, a new germline *CHEK2* LOF mutation was found in a CRC family, which is unavailable in the gnomAD database. The identified LOF mutation in *CHEK2* (*CHEK2*_p.Q27*) is predicted to cause a premature stop codon in the SQ/TQ motif, and in silico pathogenicity tools showed this mutation has the highest CADD score. We also identified another rare genetic variant (NM_001005735:c. 766 C>G, p. P256A) in *CHEK2* in 126 sporadic CRCs. These mutations in *CHEK2* may represent a genetic cause of intestinal neoplasia and cancer.

It is also known that Chk2 is activated during DNA damage and subsequently inhibits CDC25C phosphatase, preventing cells from entering mitosis, and stabilizes p53, resulting in a cell cycle arrest in G1. In our study, by performing *CHEK2* gene editing in a cellular model, we were able to demonstrate its plausible effect on cell cycle arrest and maintain genomic integrity. Consistent with previous reports, *CHEK2*^KO^ cells were unable to effectively maintain cell cycle arrest in the G2 phase with nocodazole treatment and were resistant to apoptosis after DNA damage. Lower levels of pan-p53 and phosphorylated p53 were detected in *CHEK2*^KO^ cells compared with WT cells when double-strand DNA breaks were induced. These results are consistent with the view that Chk2 plays an important role in cell cycle regulation and genomic integrity maintenance.

Loss of cell cycle checkpoint capacity caused by *CHEK2* mutant genotypes may lead to specific somatic mutations in affected colon tissues. In our study, we found *APC* somatic mutations in adenomas of *CHEK2* mutant carriers. *APC* somatic mutations have been shown to precede other germline gene mutation carriers in CRC or adenomas (7, 8, 12). *CHEK2* LOH was found in the cancer tissue of II-1, and IHC staining showed Chk2 expressed in adjacent normal cells but not in cancer cells, indicating second-hit somatic inactivation of the WT allele in cancer cells. In addition, in this pedigree, we also observed a preferential development of CRC with *TP53* mutations in germline *CHEK2* mutant carriers, where *CHEK2* second-hit inactivation may precede *TP53* mutation. In previous studies, somatic mutations in *KRAS*, *BRAF*, and *PIK3CA* have been found in CRC or adenomas from other germline gene mutation carriers. But in our study, somatic mutations were not found in these genes, suggesting that the tumors did not follow the pathway of colorectal tumorigenesis induced by somatic mutations in *KRAS*, *BRAF*, and *PIK3CA*. It has been reported that Chk2 can stabilize p53 during DNA damage. A possible mechanism is that the LOF Chk2 could not effectively stabilize p53.

Besides the genes related to the FA DNA repair pathway or the cell cycle, we also found another 2 genes (*CTNNB1* and *AMER1*; *CTNNB1*:c.1444C>G;p.Q482E, *AMER1*:c.3145C>T; p.R1049*; ClinVar accessions SCV001754805 and SCV001754806) related to the Wnt signaling pathway. *AMER1* is located on chromosome X, and the mutation carrier was a male patient. Therefore, the mutation in *AMER1* was a homozygous variant. It is well known that the Wnt signaling pathway is strongly associated with colorectal carcinogenesis. Although somatic mutations in *CTNNB1* occur frequently in colon cancer, germline mutations have been less implicated in hereditary CRC. This finding may indicate that germline mutations in *CTNNB1* or *AMER1* may be the candidate genes for CRC. Additionally, we also found a missense mutation in *SGK2* that took part in the PI3K-Akt signaling pathway. We also identified rare variants in *POLE* and *BRF1*, which have been reported as the pathogenic genes (7, 45). Among this, 1 patient carrying the *POLE* variant was an early-onset CRC. Another patient carried compound heterozygous mutations in *POLE*. This confirmed that *POLE* takes part in the colorectal tumorigenesis.

In summary, our results highlight some candidate genes for CRC germline predisposition, which involved in DNA repair and the cell cycle. Our findings implicate germline *CHEK2* mutations in the inherited susceptibility to CRC, as well as the defective cell cycle arrest and apoptosis, as the plausible underlying mechanism. Our results further support the relationship between DNA repair and cancer predisposition.

## Methods

### Patients.

We selected 24 individuals from 21 families. All of these individuals had strong CRC aggregation, but other known germline alterations of hereditary cancer syndromes (FAP and Lynch syndrome) tested negative. *APC* and *MUTYH* were tested by Sanger sequencing to identify FAP. Lynch syndrome was excluded by IHC to test the expression of MSH2, MSH6, PMS2, and MLH1. These individuals fulfilled the following criteria: they had 2 or more relatives with CRC or CRA, and 2 or more consecutive affected generations, and all individuals screened negative for FAP and Lynch syndrome. Family members were included for segregation analysis of genetic variants.

In order to investigate the frequency of candidate mutations in sporadic CRC/CRA and normal populations, 100 healthy controls who were of Chinese ancestry but had not been diagnosed with polyposis or CRC and 352 individuals with colorectal tumor (CRC/CRA) were recruited from Renji Hospital, School of Medicine, Shanghai Jiao Tong University, for further variant genotyping.

### DNA extractions and WES.

Peripheral-blood genomic DNA was extracted by QIAamp DNA Blood Kit (Qiagen). WES was performed in individuals, similar to previous reports with some modifications (46). In brief, whole-exome capture and library preparation were performed using the Twist Fast Hybridization target enrichment system. The captured library was sequenced on the Illumina HiSeq platform (Illumina Nova 6000) according to the manufacturer’s protocol. Subsequently, reads were trimmed and mapped to the human hg19 genome reference assembly with Burrows-Wheeler Alignment (BWA) and sorted by Picard-tools. Single-nucleotide variants (SNVs) and indel variants were called with Genome Analysis Toolkit (GATK). The variants were further annotated by ANNOVAR with 1000 Genomes Project, gnomAD, ExAC, SIFT, PolyPhen2 (http://genetics.bwh.harvard.edu/pph2/dokuwiki/overview), MutationTaster (http://www.mutationtaster.org/), CLINVAR, CADD (http://cadd.gs.washington.edu/score), COSMIC (http://cancer.sanger.ac.uk/cancergenome/projects/cosmic/), Generic mutation, GO terms (http://www.geneontology.org/), KEGG pathway (http://www.genome.jp/kegg/pathway.html).

### Bioinformatic analysis.

To identify the candidate genes, we further analyzed the results using standard filters steps: (a) First, we excluded the variants that did not pass the quality filter. (b) Then, we excluded variants that were not protein-coding or splicing sites. Synonymous variants were also excluded. (c) We excluded variants in 1000 genomes or gnomAD or ExAC at frequency ≥ 0.001 (7, 8, 10). (d) We screened the deleterious variants by ≥ 3 in silico prediction tools for missense variants or frameshift variants. (e) We then presented the functional or bibliographical terms filter. (f) We screened the known CRC susceptibility genes including *APC*, *MSH2*, and *PTEN* (Supplemental Table 11). Individuals were selected if they carried the deleterious variants among these known genes. The other individuals did not carry the known CRC germline mutation genes for next step screen. (g) Variants were screened by manual reviews using the filtering standards: rare nonsynonymous and possibly damaging variants in DDR genes or participating in cell apoptosis, autophagy, cell cycle, cell growth, cell proliferation, angiogenesis, inflammatory response, cell differentiation, cell adhesion, and chromatin modification function. (h) We filtered the prioritize variants that fulfilled previous criteria — with interesting gene function and interactions — and were located in protein domains. (i) The filtered candidate genes were listed for further analysis. The screening flow chart is shown in Supplemental Figure 3.

### Somatic mutation screening with WES.

DNA extraction from formalin-fixed paraffin-embedded tissue was performed with the QIAamp DNA FFPE Tissue Kit (Qiagen) according to manufacturer’s instructions. The methods of WES and annotations were performed as described above. *KRAS*, *BRAF*, and *FBXW7* mutations and a total of 83 cancer-related genes were screened, including *APC*, *CTNNB1*, and *PIK3CA* (Supplemental Table 9).

### Sanger sequencing of the candidate genes.

The candidate pathogenic variants were verified by Sanger sequencing. One hundred healthy controls and 352 CRC/CRAs were also tested for the candidate variant using Sanger sequencing. The entire *CHEK2* coding sequence was also performed by Sanger sequencing. Primer sequences are given in Supplemental Table 12.

### IHC.

Immunostains for Chk2 protein expression were performed on 4 μm sections from colon tumor and normal mucosa from family K and CRC control or healthy control. After deparaffinization, citrate buffer was used to retrieve antigen. Endogenous peroxidase was inactivated with 3% H_2_O_2_ dilution for 15 minutes. Tissues were blocked with 10% goat serum for 1 hour at room temperature. The Chk2 primary antibody (ab109413, diluted at 1:100, Abcam) was incubated overnight at 4°C. The goat anti-rabbit/mouse secondary antibody (Thermo Fisher Scientific, D-3004) was incubated for 1 hour at room temperature and subsequently revealed with DAB substrate (Dako) for 3 minutes. Slides were finally stained in hematoxylin. Immunostains for MSH2 (Thermo Fisher Scientific, diluted at 1:150, 33-7900), MSH6 (Abcam, diluted at 1:150, ab92471), PMS2 (Abcam, diluted at 1:100, ab110638), and MLH1 (Abcam, diluted at 1:100, ab92312) proteins expressions were performed as described above.

### Functional characterization of genetic variants.

The SW480 human CRC cell line was purchased from American Type Culture Collection (ATCC) and was cultured in 1640 medium supplemented with 10% FBS (Thermo Fisher Scientific) and 1% penicillin-streptomycin solution (MilliporeSigma). Cells were maintained in a humidified incubator adjusted with 5% CO_2_ at 37°C.

### Establishment of CRISPR-KO cells.

In order to generate *CHEK2*^KO^ mutant cells, the CRISPR-Cas9 system was used in accordance with manufacturer’s protocol. The CRISPR designing tool (http://www.rgenome.net/cas-designer/) was used to design the single guide RNA (sgRNA). The sequences were used as list in Supplemental Table 12. The sgRNA was cloned into the pSpCas9(BB)-2A-GFP (PX458) vector and transiently transfected into the SW480 CRC cell line using FuGENE HD transfection reagent (Promega). Forty-eight hours later, transfected cells were plated onto 96-well plates for single cell cloning. After 2 weeks, genomic DNA was extracted from each clone and subjected to PCR amplification. The positive clones were screened by Sanger sequencing. The expression of Chk2 of the positive clones was verified by Western blot.

### Protein extraction, Western blot, and antibodies.

Whole-cell protein extracts were prepared with RIPA buffer supplemented with complete Protease Inhibitor Cocktail (Roche Life Sciences) and quantified using BCA protein assay kit (Thermo Fisher Scientific). Equal aliquot of protein lysate was run on a Tris protein gel electrophoresis and transferred onto a PVDF membrane (MilliporeSigma), according to manufacturer’s protocols. Proteins were blocked in 5% BSA for 1 hour and blotted with the indicated primary antibodies, which were diluted with 5% BSA at 4°C overnight. Secondary antibodies were labeled and detected using ECL Kit (Pierce Biotech) by ChemiDoc Touch Imaging System (Bio-Rad) as described previously (47).

The antibodies were used as follows: anti-Chk2 (Abcam, diluted at 1:5000, ab109413); anti–phospho-γH2AX (phospho Ser139; Cell Signaling Technology [CST], diluted at 1:1000, 9718); anti-p53 (Abcam, diluted at 1:1000, ab32389); anti-p53 (phospho Ser20; Abcam, diluted at 1:1000, ab157454); and anti–β-actin (KangChen, diluted at 1:3000, KC-5A08).

### Cell proliferation.

The proliferation ability of cells was measured using the Cell Counting Kit-8 (CCK8) kit. Cells were inoculated into 96-well plates at a density of 2000 cells per well in sextuplicate. In total, 10 μL of CCK8 aqueous reagent and 90 μL 1640 median was added to each well after 24, 48, 72, or 96 hours, respectively. After incubation at 37°C for 2 hours, the absorbance was read at 450 nm with an Epoch Microplate Spectrophotometer. All experiments were repeated 3 times.

### Crystal violet assay.

Cells were inoculated into 24-well plates at a density of 2 × 10^4^ cells per well. After being treated with nocodazole (MedChenExpress, 31430-18-9) or Adriamycin (Selleck Chemicals, S1208) for 48 hours, cells were fixed with 4% formaldehyde for 20 minutes and dyed with 0.05% crystal violet for 20 minutes. All experiments were repeated 3 times. The ImageJ (NIH) software was used to calculate the normalized cell population in each well.

### DNA damage.

DNA damage was accessed in the presence of nocodazole with a concentration range from 0.8 μM to 2 μM or Adriamycin with a concentration range from 0.5 μM to 6 μM for a certain amount of time such as 24 hours or 48 hours. Cell lysates were collected and subjected to SDS-PAGE for Western blot analysis using primary antibodies.

### Apoptosis and cell cycle assays.

Cell apoptosis and cell cycle were measured after being treated with nocodazole or Adriamycin at the indicated time. Apoptotic cells were detected by labeling the samples with FITC–annexin V and propidium iodide (PI) (556547, BD Biosciences) in accordance with the manufacturer’s protocol. For cell cycle analysis, cells were washed in phosphate-buffered saline and vortexed while adding 75% ethanol drop-wise to fix them. Cells were incubated at 4°C overnight. After that, cells were centrifuged at 400*g* at 4°C for 5 minutes and resuspended in PBS containing 400 μL PI (550825, BD Biosciences). Cells were incubated at 37°C in the dark for 20 minutes before flow cytometry analysis. Samples were analyzed on a FACS Calibur flow cytometer (BD Biosciences); the FlowJo vision 10 software and the Modfit LT software were used to define apoptotic cells or number of cells in each cell cycle.

### Accession numbers.

The accession numbers for CNVs reported in this paper are ClinVar: SCV001754535, SCV001754757, SCV001754759, SCV001754761, SCV001754762, SCV001754763, SCV001754805, SCV001754806, SCV001754807, SCV001754808, and SCV001754809.

### Statistics.

Data from at least 3 independent experiments performed are presented as the mean ± SEM. Measurement of data between 2 groups was performed using nonparametric Mann–Whitney *U* test. Statistical tests were 2-tailed, and a *P* value less than 0.05 was considered statistically significant. SPSS statistical software was used for analyses. Graphs and associated statistical analyses were performed using GraphPad Prism version 8 for Windows (GraphPad Software).

### Study approval.

This study was approved by the institutional ethics committee (KY2019-007) of Renji Hospital, School of Medicine, Shanghai Jiao Tong University, as well as by the Chinese National Review Committee for Genetics Studies. A written informed consent was signed by each participant.

## Author contributions

PX contributed experimental design and conducted experiments, data analyses, and manuscript writing. DS contributed experimental design and data analyses, as well as manuscript writing. YG and YJ performed experiments. GZ, MZ, JC, ZW, and QL provided colon cancer specimens and clinical and pathological information. JH and HC discussed the data. PX, DS, and JYF conceived of the study and wrote the manuscript. YXC and JYF designed or/and supervised this project and revised the manuscript. The order of the co–first authors was based on the contributions to the work.

## Supplementary Material

Supplemental data

## Figures and Tables

**Table 1:**
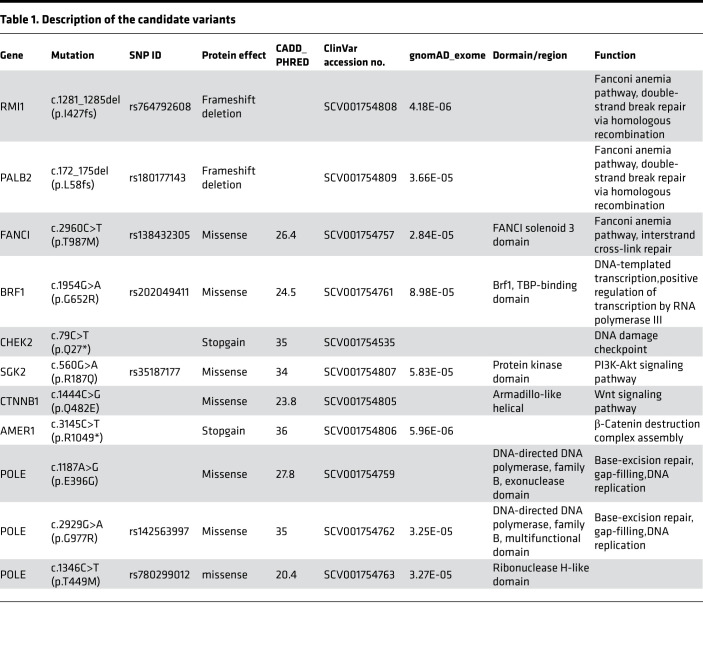
Description of the candidate variants

**Table 2:**
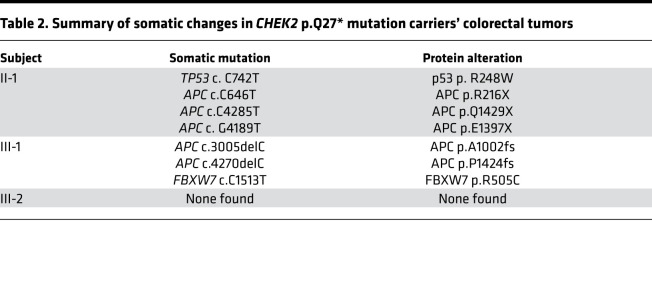
Summary of somatic changes in *CHEK2* p.Q27* mutation carriers’ colorectal tumors

## Version 1 09/22/2021

Electronic publication
