# Supplementary material for: Germline mutations in a DNA repair pathway are associated with familial colorectal cancer
Source: JCI Insight. 2021 Sep 22;6(18):e148931. doi: 10.1172/jci.insight.148931 (PMC8492347; doi:10.1172/jci.insight.148931)
Supplement: Supplemental data [file jciinsight-6-148931-s278.pdf]

SUPPLEMENTARY APPENDIX

Germline mutations in DNA repair pathway are associated with familial colorectal cancer

Pingping Xu[1†], Danfeng Sun[1†], Yaqi Gao[1], Yi Jiang[1], Ming Zhong[2], Gang Zhao[2], Jinxian Chen[2], Zheng Wang[2], Qiang Liu[3], Jie Hong[1], Haoyan Chen[1], Ying-Xuan Chen[1]*, Jing-Yuan Fang[1]*

[1]State Key Laboratory for Oncogenes and Related Genes; Key Laboratory of Gastroenterology & Hepatology, Ministry of Health; Division of Gastroenterology and Hepatology; Shanghai Institute of Digestive Disease; Renji Hospital, School of Medicine, Shanghai Jiao Tong University. 145 Middle Shandong Road, Shanghai 200001, China.
[2]Department of Surgery, Renji Hospital, School of Medicine, Shanghai Jiao Tong University, Shanghai, China
[3]Department of Pathology, Renji Hospital, School of Medicine, Shanghai Jiao Tong University, Shanghai, China

Correspondence:
Correspondence should be addressed to: JY.F: jingyuanfang@sjtu.edu.cn or YX.C: yingxuanchen71@sjtu.edu.cn

[†]P. Xu and D. Sun contributed equally to this article.

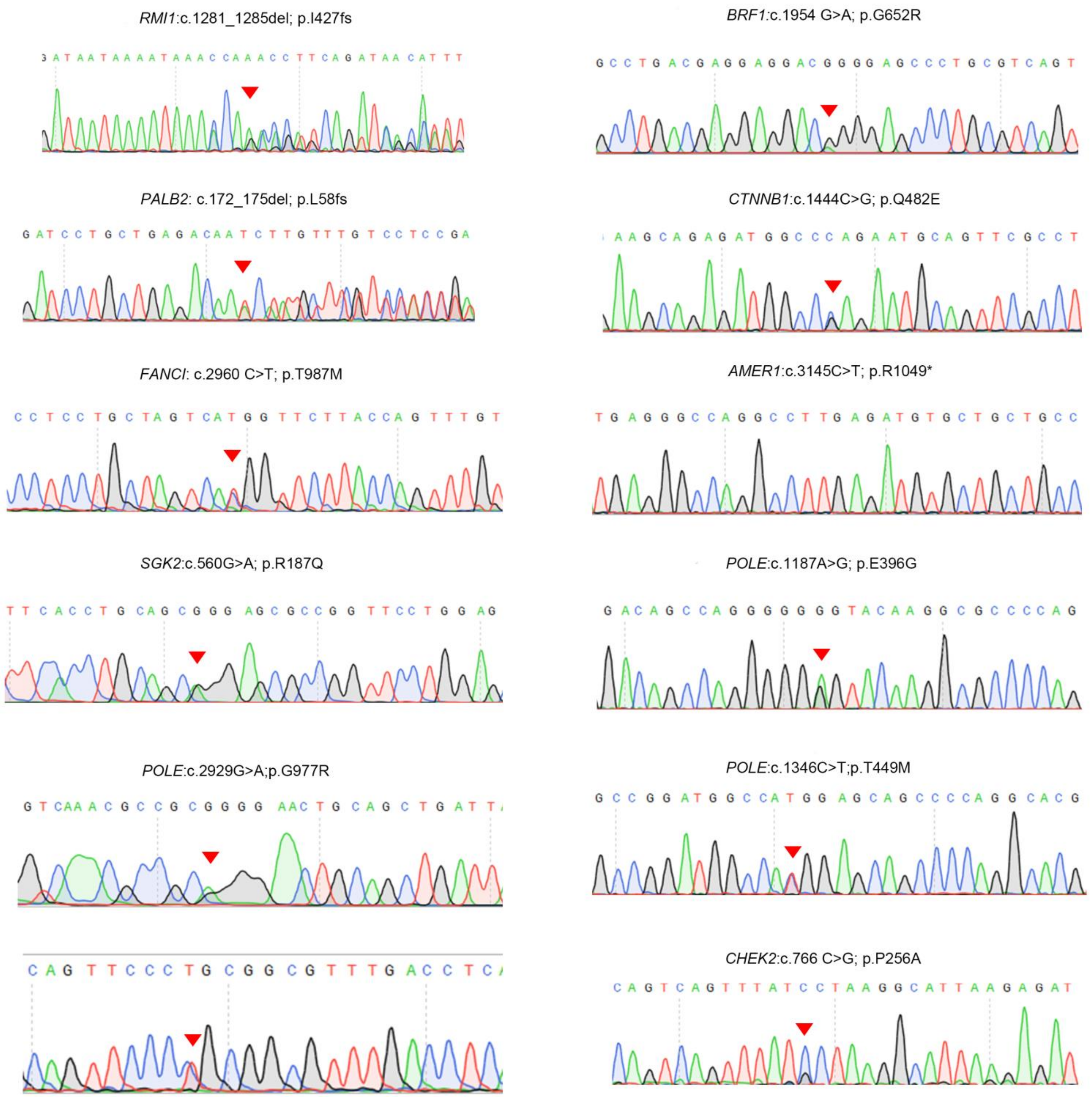

Figure S1. Sanger sequencing of mutations of interest, related to Figure1. Genomic coordinates are listed in reference to hg19. Red arrows display site of mutation.

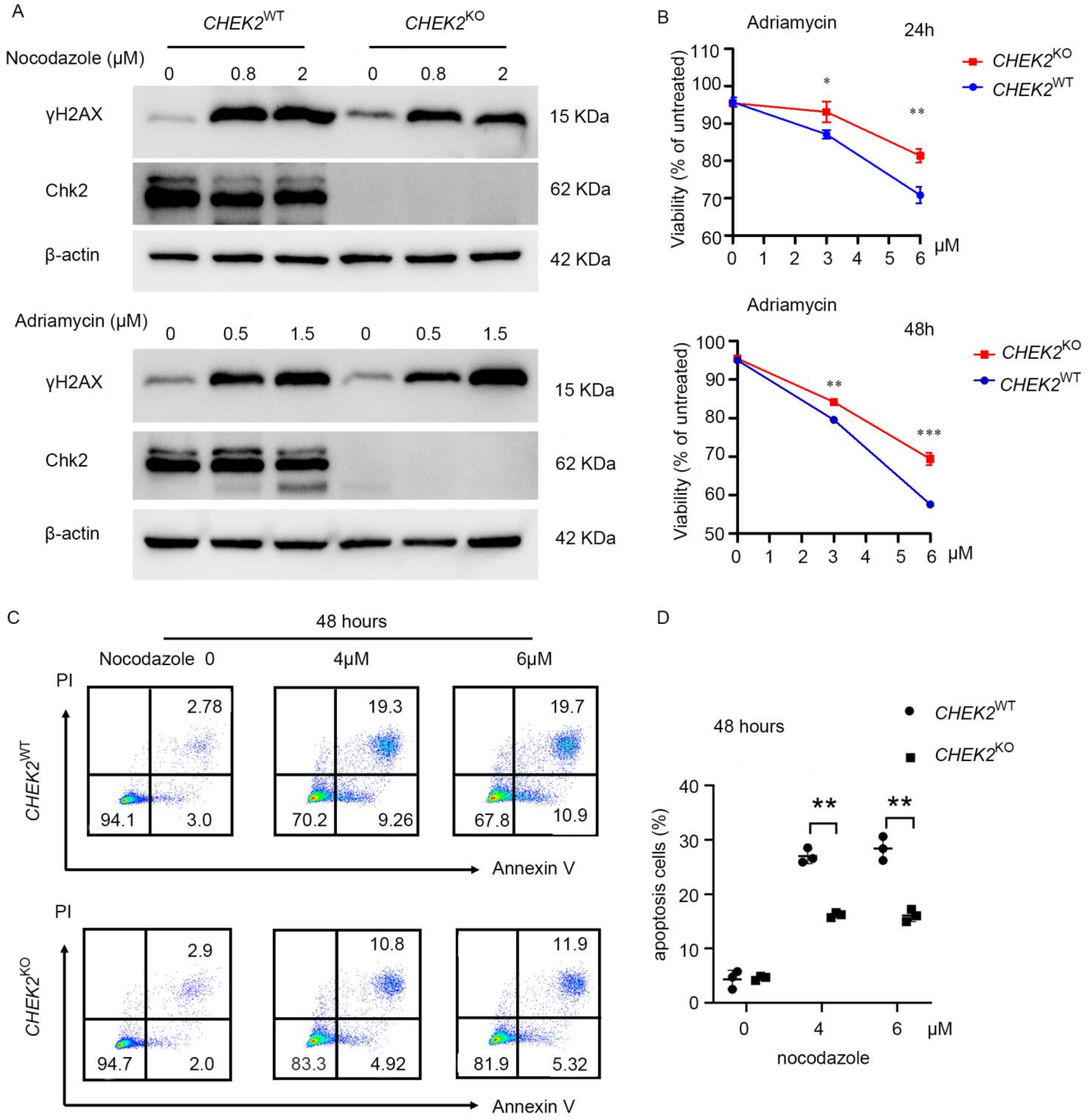

Figure S2 Failure of maintenance of nocodazole-induced G2 arrest and impaired DNA damage-induced apoptosis in *CHEK2*^{KO} cells. (A) Protein level of γH2AX, a DNA damage marker, assessed by Western blot after cells treated with nocodazole or Adriamycin. (B) The percentage of cell viability calculated by flow cytometry in *CHEK2*^{WT} and *CHEK2*^{KO} cells treated with different concentration of Adriamycin. Data are expressed as mean ± SEM (n=3, *p<0.05, **p<0.01, ***p<0.001) , nonparametric Mann–Whitney test. (C) Cellular apoptosis analysis by flow cytometry in *CHEK2*^{WT} and *CHEK2*^{KO} cells treated with different concentration of nocodazole after 48 hours. (D) The percentage of apoptosis cells were calculated by panel C. Data are expressed as mean ± SE (n=3, **p<0.01) , nonparametric Mann–Whitney test.

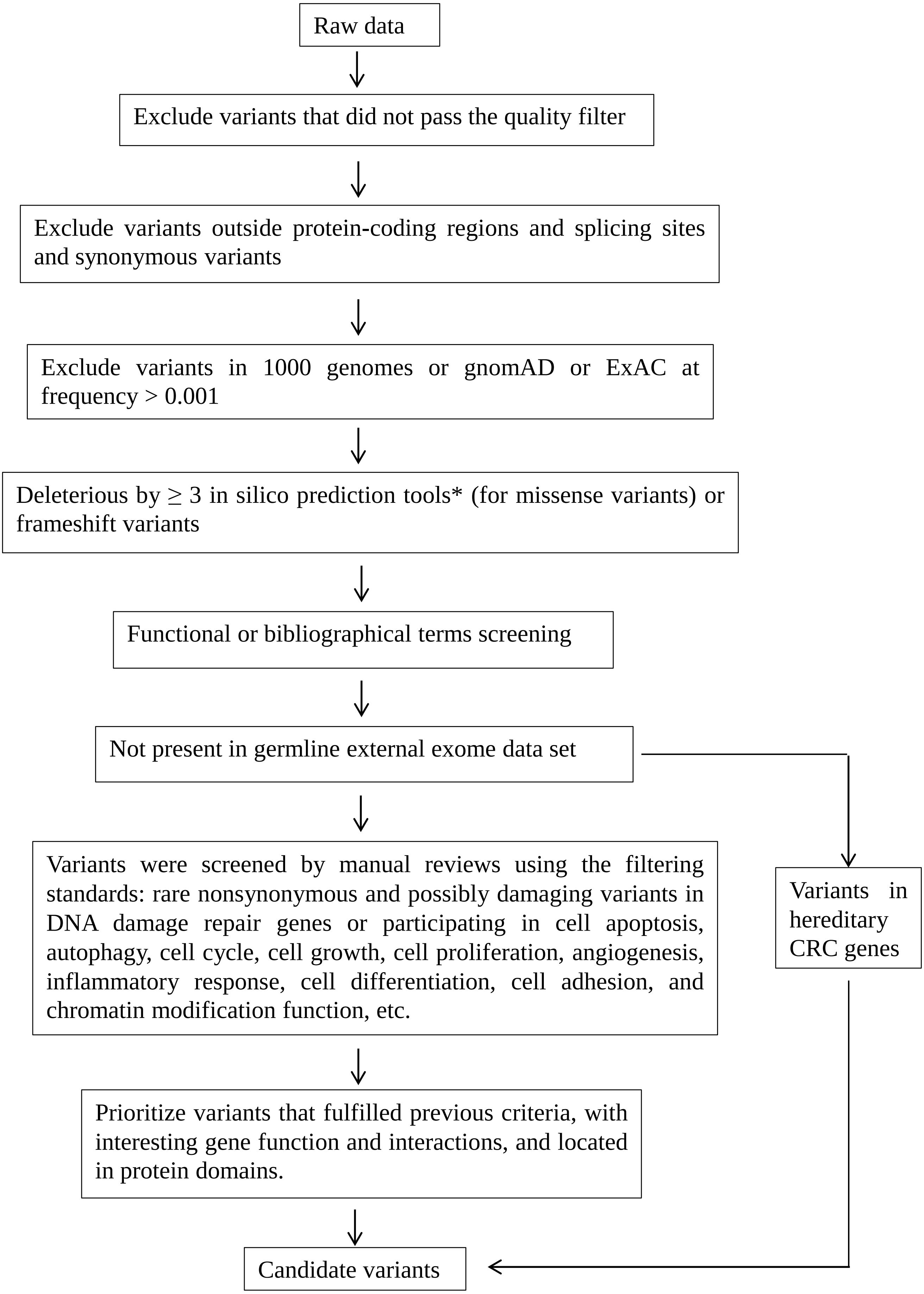

Figure S3 Initial algorithm for prioritisation of discovery phase variants for further evaluation. Twenty-four individuals with colorectal cancer (CRC) or advanced colorectal adenoma (CRA) from 21 families with CRC aggregation compatible with an autosomal dominant pattern of inheritance were sequenced. * in silico prediction tools including SIFT, PolyPhen2, MutationTaster, CLINVAR, CADD.

Supplementary Table S1. Clinical characteristics of 24 individuals included for whole-exome sequencing.

Clinical selection	Number of individuals
Number of polyps	
Less than 5 polys	10
Between 5 and 10 polys	6
Between 10 and 20 polys	6
More than 20 polys	1
Not Applicable	1
Type of polyps	
Adenomatous polyps	14
Adenomatous + hyperplastic polyps	5
Adenomatous + serrated polyps	1
Not available	4
Also diagnosed with colorectal cancer	
Yes	10
No	14
Family history FDR (first degree relative)	
Polyposis	3
CRC	6
Polyposis + CRC	15

Supplementary Table S2. Clinical characteristics of individuals included for WES

Subject	Sex	Age	Polyp type	Polyp number	Colorectal cancer	FDR with CRC or polyposis
S11	Male	72	A	12	N	Y
S12	Female	67	NA	2	Y(T3N0M0)	Y
S13	Male	64	A	5	N	Y
S15(II-1)	Male	83	A	33	Y(T4N0M0)	Y
S16(III-1)	Female	57	A,H	5	N	Y
S21	Male	63	A,S	7	N	Y
S23	Male	59	A,H	3	Y(T2N0M0)	Y
S24	Male	64	NA	0	Y(T4N2M1)	Y
S25	Male	45	A	2	N	Y
S26	Male	64	A	12	N	Y
S27	Male	72	NA	NA	Y(T4N2M0)	Y
S28	Male	62	A	10	N	Y
S29	Female	60	A	9	N	Y
S30	Female	67	A	7	N	Y
S31	Male	57	A	10	N	Y
S32	Female	24	NA	0	Y(T4N2M1)	Y
S33	Male	63	A,H	2	N	Y
S34	Male	63	A	1	N	Y
S36	Male	68	A,H	2	Y(T1N0M0)	Y
S37	Male	55	A	10	N	Y
S38	Male	65	A	7	Y(T1N0M0)	Y
S54	Female	71	A	10	Y(T1N0M0)	Y
S55	Male	39	A,H	4	N	Y
S57	Male	44	A	4	Y(T1N0M0)	Y

List is ranked based on (i) polyp type, (ii) the number of polyps present at time of diagnosis, (iii) positive history of CRC development in the index patient, and (iv) positive history of CRC or polyposis development in a first degree relative (FDR) of the index patients.

Polyp type: A: Adenomatous polyps, H: Hyperplastic polyps, S: Serrated polyps.

NA: Not Applicable.

N: no, Y: yes.

Supplementary Table S3. Overview of whole-exome sequencing statistics

Subject	NGS platform	Total Reads	% on target reads	Mean target coverage	% regions ⩾ 5X coverage	% regions ⩾ 10X coverage	% regions ⩾ 20X coverage	% regions ⩾ 30X coverage
S11	Illumina Hi Seq	51058997	66.44%	63.7	99.86	99.58	97.73	92.03
S12	Illumina Hi Seq	52876569	67.41%	66.9	99.87	99.66	98.34	93.54
S13	Illumina Hi Seq	60441143	66.65%	75.4	99.90	99.73	98.51	94.72
S15	Illumina Hi Seq	49192186	66.87%	61.6	99.85	99.52	97.64	91.10
S16	Illumina Hi Seq	60794025	67.65%	77.1	99.88	99.72	98.97	96.56
S21	Illumina Hi Seq	64984139	66.16%	80.5	99.91	99.74	98.92	96.62
S23	Illumina Hi Seq	66362828	65.82%	81.7	99.90	99.74	98.95	96.68
S24	Illumina Hi Seq	74731392	66.68%	93.3	99.93	99.82	99.30	97.95
S25	Illumina Hi Seq	75874094	67.10%	94.9	99.93	99.82	99.33	98.01
S26	Illumina Hi Seq	78058651	66.40%	97.1	99.93	99.82	99.35	98.18
S27	Illumina Hi Seq	82652899	66.72%	103.1	99.92	99.82	99.42	98.45
S28	Illumina Hi Seq	74305829	66.36%	92.4	99.93	99.81	99.26	97.86
S29	Illumina Hi Seq	71487859	66.15%	88.7	99.89	99.75	99.23	98.01
S30	Illumina Hi Seq	71361974	66.58%	88.6	99.92	99.80	99.33	98.03
S31	Illumina Hi Seq	62417198	64.99%	76.2	99.88	99.69	98.76	96.11
S32	Illumina Hi Seq	59532699	67.10%	75.1	99.89	99.70	98.88	96.40
S33	Illumina Hi Seq	54009182	66.21%	67.1	99.87	99.62	98.13	93.67
S34	Illumina Hi Seq	59240432	66.41%	73.7	99.90	99.70	98.60	95.29
S36	Illumina Hi Seq	60645027	66.14%	75.0	99.90	99.71	98.69	95.64
S37	Illumina Hi Seq	57569966	66.36%	71.8	99.89	99.69	98.51	94.94
S38	Illumina Hi Seq	53633014	66.73%	67.0	99.88	99.63	98.10	93.52
S54	Illumina Hi Seq	80739327	82.05%	131.5	98.74	98.07	96.83	95.51
S55	Illumina Hi Seq	88645809	83.45%	146.8	98.86	98.23	97.12	95.93
S57	Illumina Hi Seq	71230174	84.93%	117.2	99.23	98.69	97.5	96.08

Supplementary Table S4. Exome performance statistics for both index patients

	II-1	III-1
#reads	49192186	60794025
% on target reads	66.87%	67.65%
Mean target coverage	61.6	77.1
% Targets ≥5x	99.85	99.88
% Targets ≥15x	98.83	99.45
% Targets≥20x	97.64	98.97
% Targets ≥30x	91.1	96.56
Genetic Ancestry	Chinese	Chinese

Supplementary Table S5A. Rare non-synonymous variants in index individuals II-1 and III-1 segregating in their affected individuals after final filter step.

Sampl	Chr.	g.potision	Ref	Var	Cover	%	SNP ID	Gene	Protein	Hgvsc	Hgvsp	CADD_	1000gen	ExAC	gnomAD
e		[hg19]	.	iant	age	Var.		name	Effect			PHRED	omes		_exome
II-1	chr1	883613	C	T	42	38.10		NOC2L	Splicing	c.1558-1G>A		24	-	-	-
II-1	chr1	120491117	G	A	102	47.06	rs781871444	NOTCH2	Missense	c.C2672T	p. T891I	22.8	-	8.24E-06	4.06E-06
II-1	chr2	25457242	C	T	105	27.62	rs147001633	DNMT3A	Missense	c.G2645A	p. R882H	33	-	0.0005	0.0002
II-1	chr2	152273367	A	G	50	50		RIF1	Missense	c.A455G	p. E152G	24.5	-	-	-
II-1	chr3	125824627	C	A	63	61.90		ALDH1L1	Missense	c.G2625T	p. K875N	27.6	-	-	-
II-1	chr4	39924346	G	C	41	56.10		PDS5A	Missense	c.C550G	p. Q184E	21.6	-	-	-
II-1	chr4	71816505	A	T	32	37.5		MOB1B	Missense	c.A6T	p.E2D	20.9	-	-	8.10E-06
II-1	chr6	83074770	C	G	83	45.78		TPBG	Missense	c.C92G	p. S31W	27.3	-	-	-
II-1	chr7	120593444	G	A	48	68.75		ING3	Missense	c.G187A	p. A63T	21.9	-	-	-
II-1	chr10	50682230	C	T	81	39.50	rs760238086	ERCC6	Missense	c.G2441A	p. G814E	24.9	-	2.48E-05	1.63E-05
II-1	chr10	124380867	A	C	122	58.20	rs745860277	DMBT1	Missense	c.A5192C	p.H1731P	22.8	-	1.66E-05	1.22E-05
II-1	chr14	21862295	G	C	66	56.06	rs369825360	CHD8	Missense	c.C5659G	p. L1887V	25.1	-	-	4.09E-06
II-1	chr17	29533355	G	T	77	23.37		NF1	Missense	c.G1358T	p. G453V	23.4			
II-1	chr22	29130631	G	A	87	47.13		CHEK2	stopgain	c.C79T	p. Q27X	35			
III-1	chr1	176176009	A	G	77	40.26		RFWD2	Missense	c.T106C	p.S36P	24.2			
III-1	chr4	71816505	A	T	32	37.5		MOB1B	Missense	c.A6T	p.E2D	20.9	-	-	8.10E-06
III-1	chr14	21862295	G	C	66	56.06	rs369825360	CHD8	Missense	c.C5659G	p. L1887V	25.1	-	-	4.09E-06
III-1	chr22	29130631	G	A	87	47.13		CHEK2	stopgain	c.C79T	p. Q27X	35			

These three variants encountered in individuals Fam K II-1 and III-1 are highlighted in grey.

Supplementary Table S5B. Compiled list of rare variants segregating in the pedigree in genes with specified role in DNA repair, apoptosis, autophagy, cell cycle, angiogenesis, cell differentiation, cell adhesion, and chromatin modification function

Patient	Gene name	Hgvsc	Hgvsp	General Function-link to cell growth
II-1	NOC2L	c.1558-1G>A		It represents a novel HDAC-independent inhibitor of histone acetyltransferase. Acts as an inhibitor of histone acetyltransferase activity. Involved in the regulation of p53/TP53-dependent apoptosis.
II-1	NOTCH2	c.C2672T	p. T891I	This gene encodes a member of the Notch family. The Notch signaling network is an evolutionarily conserved intercellular signaling pathway which regulates interactions between physically adjacent cells.
II-1	DNMT3A	c.G2645A	p. R882H	This gene encodes a DNA methyltransferase that is thought to function in de novo methylation. CpG methylation is an epigenetic modification that is important for embryonic development, imprinting, and X-chromosome inactivation.
II-1	RIF1	c.A455G	p. E152G	This protein localizes to aberrant telomeres may be involved in DNA repair. Key regulator of TP53BP1 that plays a key role in the repair of double-strand DNA breaks (DSBs) in response to DNA damage.
II-1	ALDH1L1	c.G2625T	p. K875N	The encoded protein belongs to the aldehyde dehydrogenase family. Loss of function or expression of this gene is associated with decreased apoptosis, increased cell motility, and cancer progression.
II-1	PDS5A	c.C550G	p. Q184E	The protein encoded by this gene binds to the cohesin complex and associates with chromatin through most of the cell cycle. Cohesion ensures that chromosome partitioning is accurate in both meiotic and mitotic cells and plays an important role in DNA repair.
II-1	MOB1B	c.A6T	p. E2D	The protein encoded by this gene is similar to the yeast Mob1 protein. Yeast Mob1 binds Mps1p, a protein kinase essential for spindle pole body duplication and mitotic checkpoint regulation.
II-1	TPBG	c.C92G	p. S31W	This gene encodes a leucine-rich transmembrane glycoprotein that may be involved in cell adhesion. May function as an inhibitor of Wnt/beta-catenin signaling by indirectly interacting with LRP6 and blocking Wnt3a-dependent LRP6 internalization.
II-1	ING3	c.G187A	p. A63T	The protein encoded by this gene is similar to ING1, a tumor suppressor protein that can interact with TP53, inhibit cell growth, and induce apoptosis. Overexpression of this gene has been shown to inhibit cell growth and induce apoptosis.
II-1	ERCC6	c.G2441A	p. G814E	This gene encodes a DNA-binding protein that is important in transcription-coupled excision repair. The encoded protein has ATP-stimulated ATPase activity, interacts with several transcription and excision repair proteins, and may promote complex formation at DNA repair sites.
II-1	DMBT1	c.A5192C	p.H1731P	May play roles in mucosal defense system, cellular immune defense and epithelial differentiation.
II-1	CHD8	c.C5659G	p. L1887V	This gene encodes a member of the chromodomain-helicase-DNA binding protein family. This gene has been shown to function in several processes that include transcriptional regulation, epigenetic remodeling, promotion of cell proliferation, and regulation of RNA synthesis.
II-1	NF1	c.G1358T	p. G453V	This gene product appears to function as a negative regulator of the ras signal transduction pathway.
II-1	CHEK2	c.C79T	p. Q27X	The protein encoded by this gene is a cell cycle checkpoint regulator and putative tumor suppressor. It is required for checkpoint-mediated cell cycle arrest, activation of DNA repair and apoptosis in response to the presence of DNA double-strand breaks.
III-1	RFWD2	c.T106C	p.S36P	Directly involved in p53 (TP53) ubiquitination and degradation, thereby abolishing p53-dependent transcription and apoptosis.
III-1	MOB1B	c.A6T	p.E2D	The protein encoded by this gene is similar to the yeast Mob1 protein. Yeast Mob1 binds Mps1p, a protein kinase essential for spindle pole body duplication and mitotic checkpoint regulation.
III-1	CHD8	c.C5659G	p. L1887V	This gene encodes a member of the chromodomain-helicase-DNA binding protein family. This gene has been shown to function in several processes that include transcriptional regulation, epigenetic remodeling, promotion of cell proliferation, and regulation of RNA synthesis.
III-1	CHEK2	c.C79T	p. Q27X	The protein encoded by this gene is a cell cycle checkpoint regulator and putative tumor suppressor. It is required for checkpoint-mediated cell cycle arrest, activation of DNA repair and apoptosis in response to the presence of DNA double-strand breaks.

Supplementary Table S6. Variants filtering strategy in individuals II-1 and III-1

Filtering step	II-1	II-2	Overlapping genes
Total variants	86846	88187	NA
Coding variants or canonical splice site	22324	22467	NA
Non synonymous variants*	11070	11141	NA
Rare (<0.1% population frequency) **	273	247	121
Predicted LOF variants or highly conserved variants ***	154	128	67
Variants in genes with previous link to cell growth ****	14	4	3

* Missense, nonsense, splicing, frameshift, codon insertion or deletion

** Population frequency of less than 0.1% in the following databases: 1000genomes, Exome Aggregation Consortium (ExAC), gnomAD_exome.

***InterVar(automated): not Likely benign or benign or CADD_phred >20; LOF, loss of function.

****GO term contains cell growth. The variants from the final filtering steps are summarized in Table S5A and GeneCards terms are presented in Table S5B.

Supplementary Table S7. Overview of whole-exome sequencing statistics of tumors

Subject	NGS platform	Total Reads	% on target reads	Mean target coverage	% regions ⩾ 5X coverage	% regions ⩾ 10X coverage	% regions ⩾ 20X coverage	% regions ⩾ 30X coverage
L11	Illumina Hi Seq	128667176	86.93%	219.1	99.17	98.55	97.49	96.43
L12	Illumina Hi Seq	55644893	81.13%	124.2	98.94	98.2	96.8	95.23
L13	Illumina Hi Seq	169301734	87.02%	292.8	99.43	99.06	98.45	97.85
L14	Illumina Hi Seq	63477732	83.13%	144.1	99.22	98.72	97.72	96.53
L15	Illumina Hi Seq	158805568	88.31%	285	99.08	98.58	97.8	97.09
L16	Illumina Hi Seq	269297080	86.2%	467.6	99.26	98.88	98.36	97.94
L17	Illumina Hi Seq	86108984	74.11%	180.9	99.18	98.75	97.91	96.78

Supplementary Table S8. Details of somatic changes in *CHEK2* p.Q27* mutation carriers' colorectal tumors

Subject	Phenotype	Block code	Tumor type	LOH CHEK2	MSI	APC	CTNNB1	KRAS	BRAF	PIK3CA	FBXW7	TP53	POLD1	POLE
II-1	CRC CRA	L17	Carcinoma	LOH§	MSS	WT	WT	WT	WT	WT	WT	p. R248W (C>T)	WT	WT
		L11	Adenoma	No LOH	MSS	p. R216X (C>T)	WT	WT	WT	WT	WT	WT	WT	WT
		L13	Adenoma	No LOH	MSS	p.Q1429X(C>T)	WT	WT	WT	WT	WT	WT	WT	WT
		L14	Adenoma	No LOH	MSS	p.E1397X(G>T)	WT	WT	WT	WT	WT	WT	WT	WT
		L15	Adenoma	No LOH	MSS	WT	WT	WT	WT	WT	WT	WT	WT	WT
III-1	CRA	L16	Adenoma	No LOH	MSS	p.A1002fs (del C) p.P1424fs (del C)	WT	WT	WT	WT	p.R505C (C>T)	WT	WT	WT
III-2	CRA	L12	Adenoma	No LOH	MSS	WT	WT	WT	WT	WT	WT	WT	WT	WT

§LOH, loss of heterozygosity，*CHEK2* c.921_922 ins ACATAAAGATCATCAGCAAAAGGAAG TTTGCTATTGGTTCAGCAAGAGAGGCA, p.D308_P309 delins TX.

Supplementary Table S9 Genes for somatic mutations screening

Genes for somatic mutations						
KRAS	*BRAF*	*TP53*	*PIK3CA*	*CTNNB1*	*FBXW7*	*CHEK2*
APC	*MLH1*	*MSH2*	*MSH6*	*PMS2*	*MUTYH*	*BMPR1A*
BMP4	*PTPRJ*	*GALNT12*	*EPHB2*	*AXIN2*	*UNC5C*	*GREM1*
STK11	*SMAD4*	*PTEN*	*KLLN*	*POLE*	*POLD1*	*AKT1*
PIK3CA	*ENG*	*BUB1*	*BUB3*	*PMS1*	*CENPE*	*KIF23*
FANCM	*LAMB4*	*PTCHD3*	*LAMC3*	*TREX2*	*NOTCH3*	*UACA*
SFXN4	*TWSG1*	*PSPH*	*NUDT7*	*ZNF490*	*PRSS37*	*CCDC18*
PRADC1	*MRPL3*	*AKR1C4*	*RNF43*	*ATM*	*PIF1*	*TELO2*
XAF1	*RBL1*	*FAF1*	*NTHL1*	*SMAD7*	*EIF3H*	*C11orf93*
CDH1	*RHPN2*	*DUSP10*	*DIP2B*	*LAMA5*	*BMP2*	*TERC*
TERT	*CDKN1A*	*POLD3*	*SHROOM2*	*SLC5A9*	*DUSP4*	*CCND2*
PITX1	*HAO1*	*NABP1*	*LAMC1*	*TBX3*	*MYC*	

Supplementary Table S10. Clinical information for variant genotyping cohort

	NO.	Sex ratio (Male: Female)	Age (mean±SD, range,) (Age)
Health control	100	61:39	49.83±9.79 （28-74）
CRA	122	83:39	58.16±11.05 （26-82）
CRC	230	138:92	63.50±10.46 (25-82)

Supplementary Table S11. Hereditary genes and GWAS hits for colorectal cancer

Hereditary genes and GWAS hits for colorectal cancer						
APC	*MLH1*	*MSH2*	*MSH6*	*PMS2*	*MUTYH*	*BMPR1A*
BMP4	*PTPRJ*	*GALNT12*	*EPHB2*	*AXIN2*	*UNC5C*	*GREM1*
STK11	*SMAD4*	*PTEN*	*KLLN*	*POLE*	*POLD1*	*AKT1*
PIK3CA	*ENG*	*BUB1*	*BUB3*	*PMS1*	*CENPE*	*KIF23*
FANCM	*LAMB4*	*PTCHD3*	*LAMC3*	*TREX2*	*NOTCH3*	*UACA*
SFXN4	*TWSG1*	*PSPH*	*NUDT7*	*ZNF490*	*PRSS37*	*CCDC18*
PRADC1	*MRPL3*	*AKR1C4*	*RNF43*	*ATM*	*PIF1*	*TELO2*
XAF1	*RBL1*	*FAF1*	*NTHL1*	*SMAD7*	*EIF3H*	*C11orf93*
CDH1	*RHPN2*	*DUSP10*	*DIP2B*	*LAMA5*	*BMP2*	*TERC*
TERT	*CDKN1A*	*POLD3*	*SHROOM2*	*SLC5A9*	*DUSP4*	*CCND2*
PITX1	*HAO1*	*NABP1*	*LAMC1*	*TBX3*	*MYC*	

Supplementary Table S12. Primers used in the different studies

	Forward Primer (5'-3')	Reverse Primer (5'-3')	Amplicon size
CHEK2 Sequencing (Sanger sequencing)			
exon 2	GCTGTTGTAATAGGCCCATCATTTAC	ACCTTCCACCTGGTAATACAACTT	490
exon 3	GGATTACATGCTTATGCCACCATG	TTCACTATGTTGGTCAGGATGG	703
exon 4-5	ACGTTTGATACATGAAATTCAACAGC	AATTTTCCTCCTATGAGAGAGTGG	507
exon 6	CCTGTTGTAAATCTGCATGGGC	AGCGCAACCCCAACTCT	790
exon 7	CTGACCTCTCGGATACGTAG	AAACAATCTTGTAACGACTTTGA	714
exon 8	TCAGGCAGCCTTGAGTCAAC	CAGCTAAATGACAGCTAGGC	625
exon 9	TCTCCCCTGATTGAACTTCCAAGC	CCAGGATGAGAAAGGCAAGCCTAC	307
exon 10	TAAAGTTCCCCAGGTGCCTC	CCACGGTCCCTCGATTTCTG	417
exon 11	TGGTTGTGTGTATCACGGCT	GAAAGGCAGCGCATGTGATT	761
exon 12	AGCCTACGTGTCTTCTTGGAC	GCATGGTGGTGTGCATCTG	448
exon 13	GTGCTGGGATTACAAGCCTAAGG	GAAACTCCCACCACAGCACATACAC	352
exon 14	CCTGTCTGCTGACTCCGTGCT	GATACCCCCCATACATCTAAAACAATTAGCT	352
exon 15	TAGACTCCTCTGGGAAGGTAGAGAC	ATCTTTGCTTATCAGCTCCTTAAGCCGA	327
exon 16	GTGGTGAGGACTCAGTTGTCAATGATGAGA	AGCCTGCACCACTGCACTTCATTCAGTC	522
Sanger validation for *CHEK2* c.79C>T			
	CGTGATGTCTCGGGAGTCG	CTGAAGGGCCCATAATCGAG	304
CHEK2 CRISPR/Cas9 vector			
sgRNA-3	caccgCTGGTAAAAATAGAGCTTGC	aaacGCAAGCTCTATTTTTACCAGc	
editing verification	TAAAGTTCCCCAGGTGCCTC	CCACGGTCCCTCGATTTCTG	417
Gene validation sequencing (Sanger sequencing)			
RMI1	AACAGAAATGCCGATCGAAGT	TGCAGTACCATCAGACACCTTT	594
PALB2	AGACACTAGCCCGCCTTCA	ATTGCTAGTCATTATCTTCACACTGTG	342
FANCI	GCCTTCCTTTCTCAGAGGTCC	AGTATGCTGTGGTAGACGCA	325
BRF1	TCTGTTGAGATACACAGGGCTC	GCTGATGCCTTTATTTCCCTTGAA	283
SGK2	GCAGCCACCCAATAACTCCT	CTCGGAGGAGGAGACTCCAA	297
CTNNB1	GTTCAGCTTCTGGGTTCAGATGAT	CTTAGACAGCCATCCAACAGC	387
AMER1	CCAGCCAGTCTCCATATAGGC	GGCTTGGCCTGTGGTAGAG	280
POLE	TGTCGACTTAGGAGGTGGCT	CAGATCTCGCTCACGGACAG	312
POLE	CCACTGCTGTGCTTGCCTT	TGAGGGTGGAGGGTAGGCC	254
POLE	TCCTTCCAGGTGGGTGAAGAG	GACAAAACACGTGTGTCCCG	315

Supplementary Table S13. Features of individuals included for whole-exome sequencing in Figure 1

Family number	Subject	Sex	Age at presentation	Age at last investigation	Adenomas to date	CRC	Mutations found	Family history of colorectal tumors
Fam A	S26	Male	49	64	12	No	*RMI1*:c.1281_1285del;p.I427fs	M CRC@ 53yo; Si 5 ads @55yo (see Figure 1A)
Fam B	S28	Male	58	62	10	No	*PALB2*: c.172_175del; p.L58fs	F CRC@ 71yo; Si 6 ads @58yo; B GC @ 57yo; N 2 ads @ 32yo; N 2 ads @ 35yo; (see Figure 1B)
Fam C	S33	Male	59	63	2	No	*FANCI*: c.2960C>T; p.T987M	F CRC@ 76yo; **B 1 ads @63yo (S34)**; B CRC @ 59yo (see Figure 1C)
Fam D	S32	Female	24	24	NA	Y(T4N2M1)	*POLE*:c.1187A>G; p.E396G	GM CRC@ 59yo; M 4 ads @ 58yo (see Figure 1D)
Fam E	S12	Female	62	67	2	Y(T3N0M0)	*BRF1*:c.1954G>A; p.G652R	F GC @ 70yo; B CRC @ 53yo; B 12 ads @ 65yo; **B 5 ads @ 56yo (S13)**; A CRC@ 56yo; C CRC @ 65yo; C CRC @ 58yo; N 2 ads @ 37yo (see Figure 1E)
Fam F	S57	Male	44	44	4	Y(T1N0M0)	*POLE*:c.2929G>A;p.G977R;c.1346C>T;p.T449M	F CRC@ 70yo; U CRC@ 62yo (see Figure 1F)
Fam G	S38	Male	63	65	7	Y(T1N0M0)	*CTNNB1*:c.1444C>G;p.Q482E	F CRC@ 63yo; B 5 ads @ 57yo (see Figure 1G)
Fam H	S36	Male	55	68	2	Y(T1N0M0)	*AMER1*:c.3145C>T; p.R1049*	F CRC@ 70yo; Si CRC@ 60yo (see Figure 1H)
Fam I	S21	Male	58	63	7	No	*SGK2*:c.560G>A; p.R187Q	M CRC@ 71yo; B 4 ads @ 57yo (see Figure 1I)
Fam K	S16	Female	50	57	5	No	*CHEK2*:c.79C>T; p.Q27*	**F CRC+25 ads @ 73yo (S15)**; B 5 ads @ 49yo (see Figure 2A)

In the family history, M=mother, F=father, Si=sister, B=brother, N= niece or nephew, U=uncle, N=niece or nephew, A=aunt, C=first cousin, GM=grandmother, ads=adenomas, CRC=colorectal cancer, CRA=colorectal adenoma, GC=gastric cancer, yo=years old at initial presentation. Individuals shown in bold were additional relatives who underwent whole-exome sequencing.
